# Supplementary material for: Age‐related changes in P2Y receptor signalling in mouse cochlear supporting cells
Source: J Physiol. 2023 Sep 16;601(19):4375–95. doi: 10.1113/JP284980 (PMC10952729; doi:10.1113/JP284980)
Supplement: Supplementary file 1 — Statistical Summary Document [file TJP-601-4375-s002.docx]

**Manuscript Title: Age-related changes in P2Y receptor signalling in the mouse cochlear supporting cells**

**Authors:** Sarah A Hool, Jing-Yi Jeng, Dan Jagger, Walter Marcotti, and Federico Ceriani

**Animal model used, if applicable:**

Mouse: C57BL/6N (**6N**); C57BL/6N^Cdh23+^ (**6N-Repaired**); C3H/HeJ (**C3H**).

**Underlying hypothesis:**

This study investigates the hypothesis that the supporting cells of the cochlea contribute to the progression of age-related hearing loss.

**Definitions of ‘n’:**

Question 1-3: n = number of animals

Question 4-13: n =number of ROIs/cells

**Mice were from both sexes unless otherwise stated**

**Statistical summary table:**

| Experimental question number* | Finding/ conclusion | Experimental location/ variable  e.g. cortex vs cerebellum or genotype | Mean  or Median value | | SD or MAD | | n (value) | | P | | Units | Data comparisons  e.g. WT vs KO | Statistical test | Any other variable  e.g. subjects’ age or sex | | Figure/table in which data are presented | | Comments  e.g. observation |
| --- | --- | --- | --- | --- | --- | --- | --- | --- | --- | --- | --- | --- | --- | --- | --- | --- | --- | --- |
| 1. Click ABRs | ABR threshold change with age in 6N but not in C3H. | 6N - 1 month | 32.0 | | 2.7 | | 5 | |  | | dB | ages | one-way ANOVA |  | | 1C | |  |
|  |  | 6N - 20 months | 83.9 | | 19.8 | | 9 | |  |  |  |  |  |  |  |  |  |  |
|  |  | C3H - 1 month | 35.0 | | 3.5 | | 5 | | 0.7607 | |  |  |  |  |  |  |  |  |
|  |  | C3H - 20 months | 41.9 | | 5.9 | | 8 | |  |  |  |  |  |  |  |  |  |  |
| 1. Pure-tone ABR thresholds | Highly elevated in 6N mice. | 6N – 1 month  3 kHz | 73.0 | | 2.7 | | 5 | | <0.0001 | | dB | 1 month  *vs*  20 months | two-way ANOVA |  | | 1D | |  |
|  |  | 6N – 1 month  6 kHz | 41.0 | | 4.2 | | 5 | |  |  |  |  |  |  |  |  |  |  |
|  |  | 6N – 1 month  12 kHz | 21.0 | | 5.5 | | 5 | |  |  |  |  |  |  |  |  |  |  |
|  |  | 6N – 1 month  18 kHz | 29.0 | | 6.55 | | 5 | |  |  |  |  |  |  |  |  |  |  |
|  |  | 6N – 1 month  24 kHz | 32.0 | | 4.5 | | 5 | |  |  |  |  |  |  |  |  |  |  |
|  |  | 6N – 1 month  30 kHz | 43.0 | | 4.5 | | 5 | |  |  |  |  |  |  |  |  |  |  |
|  |  | 6N – 1 month  36 kHz | 62.0 | | 17.2 | | 5 | |  |  |  |  |  |  |  |  |  |  |
|  |  | 6N – 1 month  42 kHz | 68.0 | | 21.7 | | 5 | |  |  |  |  |  |  |  |  |  |  |
|  |  | 6N – 20 month  3 kHz | 93.9 | | 8.9 | | 9 | |  |  |  |  |  |  |  |  |  |  |
|  |  | 6N – 20 months  6 kHz | 85. 6 | | 10.4 | | 9 | |  |  |  |  |  |  |  |  |  |  |
|  |  | 6N – 20 months  12 kHz | 76.1 | | 22.3 | | 9 | |  |  |  |  |  |  |  |  |  |  |
|  |  | 6N – 20 months  18 kHz | 86.1 | | 14.7 | | 9 | |  |  |  |  |  |  |  |  |  |  |
|  |  | 6N – 20 months  24 kHz | 100.0 | | 0 | | 9 | |  |  |  |  |  |  |  |  |  |  |
|  |  | 6N – 20 months  30 kHz | 100.0 | | 0 | | 9 | |  |  |  |  |  |  |  |  |  |  |
|  |  | 6N – 20 months  36 kHz | 100.0 | | 0 | | 9 | |  |  |  |  |  |  |  |  |  |  |
|  |  | 6N – 20 months  42 kHz | 99.4 | | 1.7 | | 9 | |  |  |  |  |  |  |  |  |  |  |
| 1. Pure-tone ABR thresholds | Slightly elevated in C3H mice. | C3H – 1 month  3 kHz | 83.0 | | 2.7 | | 5 | | 0.0155 | | dB | 1 month  *vs*  20 months | two-way ANOVA |  | | 1E | |  |
|  |  | C3H – 1 month  6 kHz | 49.0 | | 2.2 | | 5 | |  |  |  |  |  |  |  |  |  |  |
|  |  | C3H – 1 month  12 kHz | 21.0 | | 2.2 | | 5 | |  |  |  |  |  |  |  |  |  |  |
|  |  | C3H – 1 month  18 kHz | 27.0 | | 2.7 | | 5 | |  |  |  |  |  |  |  |  |  |  |
|  |  | C3H – 1 month  24 kHz | 31.0 | | 4.2 | | 5 | |  |  |  |  |  |  |  |  |  |  |
|  |  | C3H – 1 month  30 kHz | 46.0 | | 6.5 | | 5 | |  |  |  |  |  |  |  |  |  |  |
|  |  | C3H – 1 month  36 kHz | 46.0 | | 5.5 | | 5 | |  |  |  |  |  |  |  |  |  |  |
|  |  | C3H – 1 month  42 kHz | 52.0 | | 5.7 | | 5 | |  |  |  |  |  |  |  |  |  |  |
|  |  | C3H – 20 months  3 kHz | 85.6 | | 7.8 | | 8 | |  |  |  |  |  |  |  |  |  |  |
|  |  | C3H – 20 months  6 kHz | 70.6 | | 6.28 | | 8 | |  |  |  |  |  |  |  |  |  |  |
|  |  | C3H – 20 months  12 kHz | 33.8 | | 7.98 | | 8 | |  |  |  |  |  |  |  |  |  |  |
|  |  | C3H – 20 months  18 kHz | 26.9 | | 6.58 | | 8 | |  |  |  |  |  |  |  |  |  |  |
|  |  | C3H – 20 months  24 kHz | 46.5 | | 11.6 | | 8 | |  |  |  |  |  |  |  |  |  |  |
|  |  | C3H – 20 months  30 kHz | 55.6 | | 15.5 | | 8 | |  |  |  |  |  |  |  |  |  |  |
|  |  | C3H – 20 months  36 kHz | 55.0 | | 12.0 | | 8 | |  |  |  |  |  |  |  |  |  |  |
|  |  | C3H – 20 months  42 kHz | 48.8 | | 14.3 | | 8 | |  |  |  |  |  |  |  |  |  |  |
| Average Ca^2+^ responses to ATP application | Change with age | 6N – P7-P8 | 0.070 | | 0.049 | | 212 | | <0.0001 | | ΔR/R0 (a.u.) | Age | Pairwise Wilcoxon rank-sum test | Strain | | 6O | |  |
|  |  | 6N – 1-2 months | 0.005 | | 0.018 | | 144 | |  |  |  |  |  |  |  |  |  |  |
|  |  | 6N – 6 months | 0.011 | | 0.011 | | 130 | |  | |  |  |  |  |  |  |  |  |
|  |  | 6N – 12-13 months | 0.045 | | 0.027 | | 134 | | <0.0001 | |  |  |  |  |  |  |  |  |
|  |  | 6N – 18-24 months | 0.100 | | 0.063 | | 276 | |  |  |  |  |  |  |  |  |  |  |
|  |  | C3H – P7-P8 | 0.079 | | 0.031 | | 127 | | <0.0001 | |  |  |  |  |  |  |  |  |
|  |  | C3H – 1-2 months | 0.002 | | 0.012 | | 194 | |  |  |  |  |  |  |  |  |  |  |
|  |  | C3H – 12-13 months | 0.037 | | 0.027 | | 127 | | >0.9999 | |  |  |  |  |  |  |  |  |
|  |  | C3H – 18-24 months | 0.040 | | 0.034 | | 173 | |  |  |  |  |  |  |  |  |  |  |
|  |  | 6N-Rep – P7-P8 | 0.070 | | 0.047 | | 176 | | <0.0001 | |  |  |  |  |  |  |  |  |
|  |  | 6N-Rep – 1-2 months | 0.017 | | 0.020 | | 171 | |  |  |  |  |  |  |  |  |  |  |
|  |  | 6N-Rep – 12-13 months | 0.056 | | 0.027 | | 88 | | <0.0001 | |  |  |  |  |  |  |  |  |
|  |  | 6N-Rep – 18-24 months | 0.101 | | 0.060 | | 250 | |  |  |  |  |  |  |  |  |  |  |
|  |  | Additional statistical comparison | | | | | | | <0.0001 | |  | 6N *vs* C3H | Tukey’s post-test | Strain | |  |  |  |
|  |  |  |  |  |  |  |  |  | 0.7176 | |  | 6N *vs* Repaired |  |  |  |  |  |  |
|  |  |  |  |  |  |  |  |  | 0.1647 | |  | P7-P8 6N *vs* C3H | Pairwise Wilcoxon rank-sum test |  |  |  |  |  |
|  |  |  |  |  |  |  |  |  | <0.0001 | |  | 18-24 mo. 6N *vs* C3H |  |  |  |  |  |  |
|  |  |  |  |  |  |  |  |  | 0.1246 | |  | 6N 1 mo. *vs* 6 mo. |  |  |  |  |  |  |
| Maximal Ca^2+^ responses to ATP application | Change with age | 6N – P7-P8 | 0.189 | | 0.143 | | 212 | | <0.0001 | | ΔR/R0 (a.u.) | Age | Pairwise Wilcoxon rank-sum test | Strain | | 6P | |  |
|  |  | 6N – 1-2 months | 0.036 | | 0.034 | | 144 | |  |  |  |  |  |  |  |  |  |  |
|  |  | 6N – 6 months | 0.034 | | 0.028 | | 130 | |  | |  |  |  |  |  |  |  |  |
|  |  | 6N – 12-13 months | 0.111 | | 0.07 | | 134 | | <0.0001 | |  |  |  |  |  |  |  |  |
|  |  | 6N – 18-24 months | 0.235 | | 0.148 | | 276 | |  |  |  |  |  |  |  |  |  |  |
|  |  | C3H – P7-P8 | 0.189 | | 0.075 | | 127 | | <0.0001 | |  |  |  |  |  |  |  |  |
|  |  | C3H – 1-2 months | 0.02 | | 0.023 | | 194 | |  |  |  |  |  |  |  |  |  |  |
|  |  | C3H – 12-13 months | 0.085 | | 0.052 | | 127 | | >0.9999 | |  |  |  |  |  |  |  |  |
|  |  | C3H – 18-24 months | 0.1 | | 0.083 | | 173 | |  |  |  |  |  |  |  |  |  |  |
|  |  | 6N-Rep – P7-P8 | 0.169 | | 0.092 | | 176 | | <0.0001 | |  |  |  |  |  |  |  |  |
|  |  | 6N-Rep – 1-2 months | 0.045 | | 0.036 | | 171 | |  |  |  |  |  |  |  |  |  |  |
|  |  | 6N-Rep – 12-13 months | 0.148 | | 0.071 | | 88 | | <0.0001 | |  |  |  |  |  |  |  |  |
|  |  | 6N-Rep – 18-24 months | 0.24 | | 0.151 | | 250 | |  |  |  |  |  |  |  |  |  |  |
|  |  | Additional statistical comparison | | | | | | | <0.0001 | |  | 6N *vs* C3H | Tukey’s post-test | Strain | |  |  |  |
|  |  |  |  |  |  |  |  |  | >0.9999 | |  | 6N *vs* Repaired |  |  |  |  |  |  |
|  |  |  |  |  |  |  |  |  | >0.9999 | |  | P7-P8 6N *vs* C3H | Pairwise Wilcoxon rank-sum test |  |  |  |  |  |
|  |  |  |  |  |  |  |  |  | <0.0001 | |  | 18-24 mo. 6N *vs* C3H |  |  |  |  |  |  |
|  |  |  |  |  |  |  |  |  | >0.9999 | |  | 6N 1 mo. *vs* 6 mo. |  |  |  |  |  |  |
| Ca^2+^ Oscillation frequency | Change with age | 6N – P7-P8 | 1.243 | | 0.895 | | 60 | | <0.0001 | | Oscillations/min | Age | Pairwise Wilcoxon rank-sum test | Strain | | 6Q | |  |
|  |  | 6N – 1-2 months | 0.676 | | 0.644 | | 52 | |  |  |  |  |  |  |  |  |  |  |
|  |  | 6N – 6 months | 0.401 | | 0.238 | | 55 | |  | |  |  |  |  |  |  |  |  |
|  |  | 6N – 12-13 months | 0.68 | | 0.667 | | 55 | | <0.0001 | |  |  |  |  |  |  |  |  |
|  |  | 6N – 18-24 months | 1.715 | | 1.308 | | 48 | |  |  |  |  |  |  |  |  |  |  |
|  |  | C3H – P7-P8 | 1.336 | | 0.634 | | 66 | | <0.0001 | |  |  |  |  |  |  |  |  |
|  |  | C3H – 1-2 months | 0.101 | | 0.205 | | 71 | |  |  |  |  |  |  |  |  |  |  |
|  |  | C3H – 12-13 months | 0.542 | | 0.452 | | 65 | | 0.3742 | |  |  |  |  |  |  |  |  |
|  |  | C3H – 18-24 months | 0.582 | | 0.569 | | 56 | |  |  |  |  |  |  |  |  |  |  |
|  |  | 6N-Rep – P7-P8 | 1.464 | | 0.61 | | 66 | | <0.0001 | |  |  |  |  |  |  |  |  |
|  |  | 6N-Rep – 1-2 months | 0.302 | | 0.194 | | 65 | |  |  |  |  |  |  |  |  |  |  |
|  |  | 6N-Rep – 12-13 months | 1.042 | | 0.71 | | 77 | | 0.0318 | |  |  |  |  |  |  |  |  |
|  |  | 6N-Rep – 18-24 months | 1.133 | | 0.955 | | 132 | |  |  |  |  |  |  |  |  |  |  |
|  |  | Additional statistical comparison | | | | | | | >0.9999 | |  | 6N 1 mo. *vs* 6 mo. | Pairwise Wilcoxon rank-sum test | Strain | |  |  |  |
| Effect of thapsigargin on ATP-induced Ca^2+^ responses | Thapsigargin abolished ATP-induced responses | 6N – P7-P8 - Ctrl | 0.157 | | 0.049 | | 102 | | <0.0001 | | ΔR/R0 (a.u.) | CTRL *vs* Thapsigargin | Wilcoxon signed-rank test |  | | 8B | | Max. Ca^2+^ response |
|  |  | 6N – P7-P8 - Thapsigargin | 0.020 | | 0.021 | |  |  |  |  |  |  |  |  |  |  |  |  |
|  |  | 6N – 18-24 months - Ctrl | 0.268 | | 0.140 | | 46 | | <0.0001 | |  |  |  |  |  |  |  |  |
|  |  | 6N – 18-24 months - Thapsigargin | 0.020 | | 0.012 | |  |  |  |  |  |  |  |  |  |  |  |  |
| Average Ca^2+^ responses to ADP application | ADP-induced responses do not change with age | 6N – P7-P8 | 0.090 | | 0.048 | | 59 | |  | | ΔR/R0 (a.u.) | Age |  |  | | 9E | |  |
|  |  | 6N – 1-2 months | 0.174 | | 0.080 | | 70 | | >0.9999 | |  |  | Pairwise Wilcoxon rank-sum test |  |  |  |  |  |
|  |  | 6N – 18-24 months | 0.184 | | 0.073 | | 121 | |  |  |  |  |  |  |  |  |  |  |
|  |  | Additional statistical comparison | | | | | | |  | |  |  |  |  | |  |  |  |
| Max Ca^2+^ responses to ADP application | ADP-induced responses do not change with age | 6N – P7-P8 | | 0.194 | | 0.092 | | 59 | |  | ΔR/R0 (a.u.) | Age |  |  | 9F | |  | |
|  |  | 6N – 1-2 months | | 0.331 | | 0.164 | | 70 | | >0.9999 |  |  | Pairwise Wilcoxon rank-sum test |  |  |  |  |  |
|  |  | 6N – 18-24 months | | 0.314 | | 0.126 | | 121 | |  |  |  |  |  |  |  |  |  |
|  |  | Additional statistical comparison | | | | | | | |  |  |  |  |  |  |  |  | |

| Effect of MRS2500 on ADP-induced responses | ADP responses in aged mice are due to P2Y_1_ receptors | 6N – 18-24 months - Ctrl | 0.220 | 0.149 | 64 | <0.0001 | ΔR/R0 (a.u.) | CTRL *vs* MRS2500 | Wilcoxon signed-rank test |  | 9H |  |
| --- | --- | --- | --- | --- | --- | --- | --- | --- | --- | --- | --- | --- |
|  |  | 6N – 18-24 months – MRS2500 | 0.017 | 0.018 |  |  |  |  |  |  |  |  |
|  |  | Additional statistical comparison | | | |  |  |  |  |  |  |  |
| Average Ca^2+^ responses to UTP application | Change with age | 6N – P7-P8 | 0.146 | 0.057 | 151 |  | ΔR/R0 (a.u.) |  | Pairwise Wilcoxon rank-sum test | Strain | 10H |  |
|  |  | 6N – 1-2 months | 0.028 | 0.034 | 104 | <0.0001 |  | 6N – P7-P8 vs 1-2 mo |  |  |  |  |
|  |  | 6N – 18-24 months | 0.148 | 0.113 | 148 | <0.0001 |  | 6N – 1-2 mo *vs* 18-24 mo |  |  |  |  |
|  |  | C3H – P7-P8 | 0.13 | 0.026 | 66 |  |  |  |  |  |  |  |
|  |  | C3H – 1-2 months | 0.017 | 0.025 | 128 | <0.0001 |  | C3H – P7-P8 vs 1-2 mo |  |  |  |  |
|  |  | C3H – 18-24 months | 0.064 | 0.036 | 125 | <0.0001 |  | C3H – 1-2 mo *vs* 18-24 mo |  |  |  |  |
|  |  | Additional statistical comparison | | | | <0.0001 |  | 18-24 mo. 6N *vs* C3H | Pairwise Wilcoxon rank-sum test | Strain |  |  |
|  |  |  |  |  |  | 0.7990 |  | P7-P8 6N *vs* C3H |  |  |  |  |
| Max Ca^2+^ responses to UTP application | Change with age | 6N – P7-P8 | 0.277 | 0.105 | 151 |  | ΔR/R0 (a.u.) |  | Pairwise Wilcoxon rank-sum test | Strain | 10I |  |
|  |  | 6N – 1-2 months | 0.074 | 0.071 | 104 | <0.0001 |  | 6N – P7-P8 vs 1-2 mo |  |  |  |  |
|  |  | 6N – 18-24 months | 0.294 | 0.222 | 148 | <0.0001 |  | 6N – 1-2 mo *vs* 18-24 mo |  |  |  |  |
|  |  | C3H – P7-P8 | 0.268 | 0.064 | 66 |  |  |  |  |  |  |  |
|  |  | C3H – 1-2 months | 0.061 | 0.058 | 128 | <0.0001 |  | C3H – P7-P8 vs 1-2 mo |  |  |  |  |
|  |  | C3H – 18-24 months | 0.146 | 0.077 | 125 | <0.0001 |  | C3H – 1-2 mo *vs* 18-24 mo |  |  |  |  |
|  |  | Additional statistical comparison | | | | <0.0001 |  | 18-24 mo. 6N *vs* C3H | Pairwise Wilcoxon rank-sum test | Strain |  |  |
|  |  |  |  |  |  | >0.9999 |  | P7-P8 6N *vs* C3H |  |  |  |  |
| Effect of AR-C 118925XX on UTP-induced responses | Blocking P2Y2 receptors reduces UTP-induced responses | 6N – 18-24 months - Ctrl | 0.185 | 0.100 | 74 | <0.0001 | ΔR/R0 (a.u.) | CTRL *vs* AR-C 118925XX | Wilcoxon signed-rank test |  | 11B |  |
|  |  | 6N – 18-24 months - AR-C 118925XX | 0.040 | 0.038 |  |  |  |  |  |  |  |  |
|  |  | Additional statistical comparison | | | |  |  |  |  |  |  |  |
